# Molecular Mechanisms Underlying Vascular Remodeling in Hypertension

**DOI:** 10.31083/j.rcm2502072

**Published:** 2024-02-20

**Authors:** Xinyi Zeng, Yan Yang

**Affiliations:** ^1^Key Lab of Medical Electrophysiology of Ministry of Education and Medical Electrophysiological Key Lab of Sichuan Province, Collaborative Innovation Center for Prevention and Treatment of Cardiovascular Disease, Institute of Cardiovascular Research, Southwest Medical University, 646000 Luzhou, Sichuan, China

**Keywords:** hypertension, vascular remodeling, vascular extracellular matrix, renin-angiotensin system, ion channel, vascular resident stem cells

## Abstract

Hypertension, a common cardiovascular disease, is primarily characterized by vascular remodeling. Recent extensive research has led to significant progress in understanding its mechanisms. Traditionally, vascular remodeling has been described as a unidirectional process in which blood vessels undergo adaptive remodeling or maladaptive remodeling. Adaptive remodeling involves an increase in vessel diameter in response to increased blood flow, while maladaptive remodeling refers to the narrowing or thickening of blood vessels in response to pathological conditions. However, recent research has revealed that vascular remodeling is much more complex. It is now understood that vascular remodeling is a dynamic interplay between various cellular and molecular events. This interplay process involves different cell types, including endothelial cells, smooth muscle cells, and immune cells, as well as their interactions with the extracellular matrix. Through these interactions, blood vessels undergo intricate and dynamic changes in structure and function in response to various stimuli. Moreover, vascular remodeling involves various factors and mechanisms such as the renin-angiotensin-aldosterone system (RAS), oxidative stress, inflammation, the extracellular matrix (ECM), sympathetic nervous system (SNS) and mechanical stress that impact the arterial wall. These factors may lead to vascular and circulatory system diseases and are primary causes of long-term increases in systemic vascular resistance in hypertensive patients. Additionally, the presence of stem cells in adventitia, media, and intima of blood vessels plays a crucial role in vascular remodeling and disease development. In the future, research will focus on examining the underlying mechanisms contributing to hypertensive vascular remodeling to develop potential solutions for hypertension treatment. This review provides us with a fresh perspective on hypertension and vascular remodeling, undoubtedly sparking further research efforts aimed at uncovering more potent treatments and enhanced preventive and control measures for this disease.

## 1. Introduction

Hypertension is a significant risk factor for numerous cardiovascular, renal, and neurovascular diseases, with the frequency and mortality rates on the rise. Coronary heart disease, heart failure, atrial fibrillation, aortic valvular disease, sudden cardiac death, and abdominal aortic aneurysms, among others, have been linked to hypertension [1, 2]. Therefore, it is crucial to uncover novel mechanisms of hypertension and identify relatively effective treatment methods.

The arterial vessel wall is a complex structure and comprised of three distinct layers: the tunica externa, the tunica media and the tunica intima [3]. During the process of angiogenesis, the vessel wall closely monitors changes in its environment, integrating intercellular communication signals that ultimately affect the structure and function of blood vessels via the local production of mediators. Vascular remodeling is a crucial and positive process involving significant structural and functional changes. This process is governed by at least four cellular processes, including growth, death, migration, and synthesis or degradation of the extracellular matrix. Vascular remodeling is heavily influenced by the interaction among local growth factors, vasoactive substances, and hemodynamic stimulation, and it represents a dynamic response process directed towards hemodynamic conditions in the long term. However, it may also lead to vascular diseases and promote issues within the circulatory system [4].

Vascular structure changes can be broadly categorized as hypertrophic or non-hypertrophic remodeling. Hypertrophic remodeling involves the enlargement and proliferation of vascular smooth muscle cells (VSMCs), while non-hypertrophic remodeling involves a rearrangement of VSMCs. Remodeling can also be further categorized as inward, outward [5], or compensatory, depending on changes in the diameter of the blood vessel [6, 7]. Structural remodeling, first described by Folkow [8], is caused by various factors, such as the sympathetic nervous system (SNS) [9], renin-angiotensin-aldosterone system (RAS), extracellular matrix (ECM), endothelium-mediated mechanisms, mitochondrial dysfunction, and genetic factors. Vascular functional remodeling refers to the adaptive changes that occur in blood vessels in response to various physiological and pathological stimuli. This process involves a series of cellular and molecular events that aim to modify the structure and function of blood vessels to meet the changing demands of the body. One important aspect of vascular functional remodeling is the changes in cell growth, proliferation, and migration. These processes involve alterations in the number and size of VSMCs, endothelial cells (ECs), and fibroblasts within blood vessel walls. In addition, the regulation of vascular tone is another important aspect of vascular functional remodeling, in which the sympathetic nervous system (SNS) plays a crucial role. There exists a significant correlation between the SNS and vascular remodeling. The SNS has a vital function in promoting vascular remodeling by controlling vascular contraction and intimal thickening. However, in certain disease conditions, the excessive activation of the SNS can lead to abnormal vascular remodeling, thereby increasing the susceptibility to various cardiovascular diseases [10, 11, 12]. Changes in ion channel remodeling in vascular cells are also particularly important in this process. It is important to note that normal blood pressure is crucial in the development and differentiation of tissues and organs, achieved through shear and tensile forces. However, high blood pressure can result in abnormal biomechanical forces that cause vascular growth and development, vascular remodeling, and arterial stiffness. A recent study by Rasna Sabharwal in 2022 [13] explored the intrinsic structural plasticity of cerebral arterioles both during and after hypertension. It was discovered that during hypertension, arteriolar wall thickness, diameter, wall cavity ratio, and biostiffness undergo rapid changes that return to normal levels once blood pressure levels are reduced. However, inward remodeling occurs gradually and does not return to normal levels. Nevertheless, there is hope for improved disease outcomes in patients with hypertension [14]. Liu *et al*. [15] carried out a study to investigate the effects of long-term administration of losartan, aspirin, and atorvastatin on vascular remodeling in young spontaneously hypertensive rats (SHR). The results showed that these drugs not only have antihypertensive, anti-inflammatory, and lipid-lowering properties, but also improve vascular remodeling.

In summary, hypertension is a complex disease involving multiple pathophysiological mechanisms. By studying and exploring factors related to vascular remodeling, a better understanding of hypertension can be achieved. This article presents an extensive overview of the latest advancements in the field of ECM, RAS, and inflammation. Additionally, we explore the pivotal role played by ion channels present in blood vessels and stem cells in hypertension-induced vascular remodeling. Due to limitations in space, this review does not cover the influence of sympathetic nerves on vascular remodeling.

## 2. Causes Associated with Vascular Remodeling

**Fig. 1.**
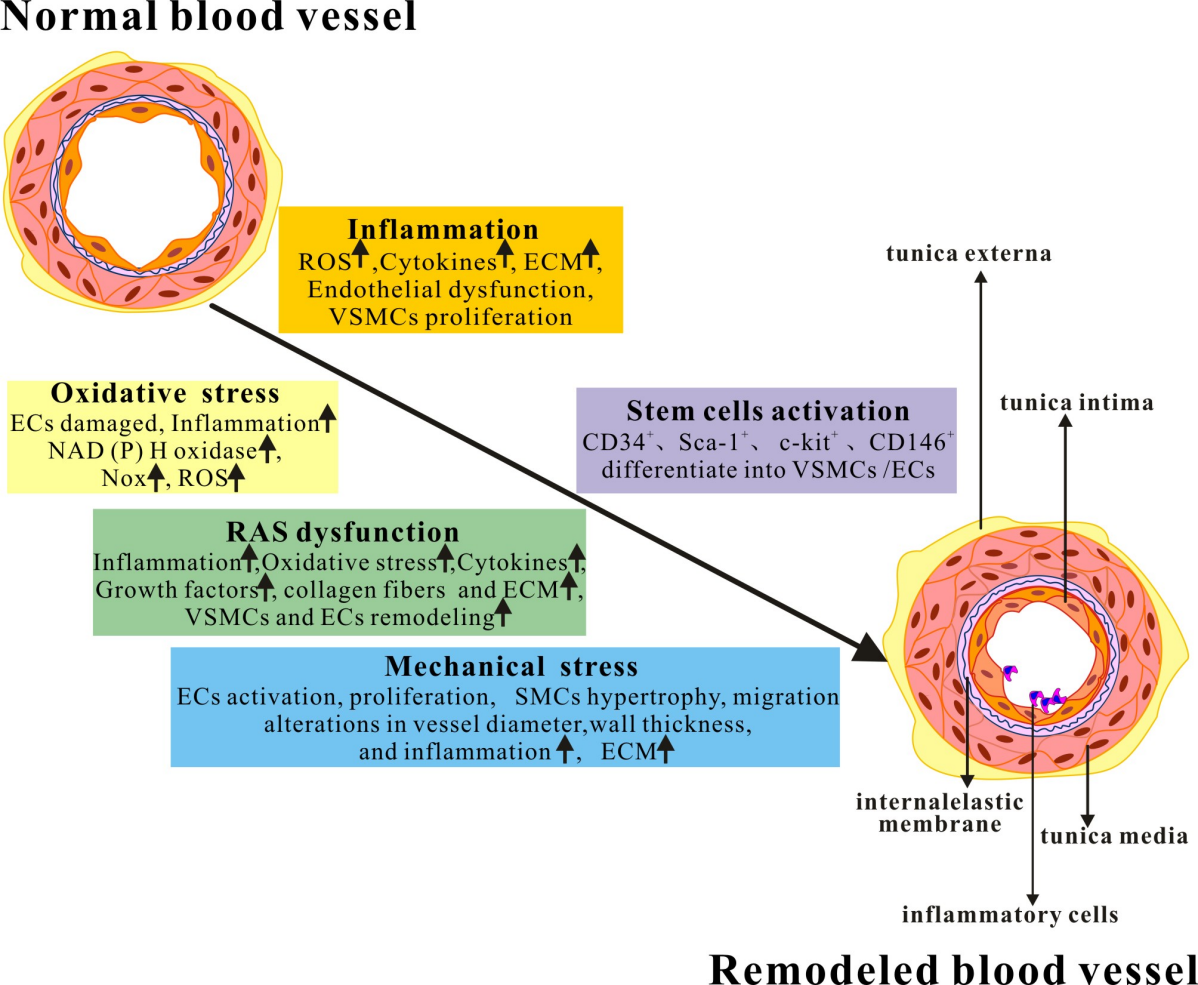
**Key factors that contribute to vascular remodeling**. The main factors and important mechanisms of vascular remodeling are comprehensively summarized in the figure. RAS, renin-angiotensin system; ROS, reactive oxygen species; ECM, extracellular matrix; NADPH oxidase, nicotinamide adenine dinucleotide phosphate oxidase; Nox, the catalytic subunit of the NADPH oxidases; ECs, endothelial cells; VSMCs, vascular smooth muscle cells; SMCs, smooth muscle cells; CD, leukocyte differentiation antigen; Sca-1, stem cell antigen-1; c-kit, stem cell growth factor receptor.

Vascular remodeling involves a multitude of factors and mechanisms, including inflammation, cytokines, RAS, and mechanical stress experienced by the arterial walls. Inflammation plays a critical role in vascular remodeling by promoting endothelial dysfunction, VSMC proliferation, and ECM deposition [16, 17]. Elevated levels of proinflammatory cytokines, such as interleukin-6 (IL-6) and tumor necrosis factor-alpha (TNF-α), are closely associated with vascular remodeling. Another important aspect of vascular remodeling is the role of matrix metalloproteinases (MMPs), which are enzymes that degrade ECM molecules [18]. Additionally, a range of cytokines can activate cellular signaling pathways inECs and VSMCs that are known to participate in the complex process of vascular remodeling. RAS plays a crucial role in regulating fluid balance and blood pressure, as well as vascular remodeling [19, 20]. The production of angiotensin II (Ang II), which is a byproduct of this system, has been proven to result in vascular remodeling. Studies suggest that Ang II pathophysiology is triggered by reactive oxygen species (ROS), which are produced via the reduced nicotinamide adenine dinucleotide phosphate oxidase (NADPH oxidase) and the catalytic subunit of the NADPH oxidases (Nox). High blood pressure can exert pressure on arterial walls, thus causing structural changes like thickening of the medial layer and deposition of collagen. Recent studies have proven that stem cells play a pivotal role in vascular remodeling. Their remarkable potential to differentiate into various cell types, such as ECs, VSMCs, and pericytes, among others, makes them a crucial component in the growth and maintenance of blood vessels. These cells are particularly involved in the formation of new vessels and repairing damaged ones. Among the numerous influencing factors, we will concentrate our attention on the latest research progress of the factors that have the most significant impact. Fig. 1 provides an overview of the key factors that contribute to vascular remodeling.

### 2.1 Vascular Extracellular Matrix (ECM) and Related Proteins

ECM is a complex network composed of various proteins that closely interact with ECs and serve important biological functions [21]. Key proteins, such as collagen, fibronectin, and MMPs, play critical roles in maintaining blood vessel structure, promoting growth factor activity, and contributing to physiological and pathological processes associated with inflammation and immune response. Angiogenesis, the formation of new blood vessels, requires the degradation of the vascular basement membrane and the remodeling of the ECM to facilitate EC migration. The ECM not only provides mechanical stability but also controls vascular cell behavior and is central to vascular function and homeostasis. ECM remodeling, including generation, degradation, and changes in arterial tissue, is a hallmark of vascular remodeling in hypertension. Extracellular protease activity and receptor cleavage are associated with hypertension-related cell dysfunction. MMPs and cathepsins play crucial roles in the remodeling of ECM, making them pivotal regulators in various biological processes. Notably, their involvement in maintaining the integrity of vascular architecture and fine-tuning growth factor signaling has garnered significant interest among researchers and scientists.

#### 2.1.1 Matrix Metalloproteinases (MMPs)

MMPs are a group of enzymes that rely on ${\mathrm{Zn}}^{2+}$ to specifically break down extracellular matrix components. They are widely expressed in most vascular cell types, indicating their potential as a source of MMP release in the ECM. Moreover, MMPs play crucial roles in both intercellular and intracellular signaling pathways in vascular cells. By 1991, seven MMPS (MMP-1, -2, -3, -7, -8, -9, and -10) were discovered, an important milestone in the understanding of the MMP family. With the completion of the human genome project, we now know that the human MMP family consists of 23 members, each possessing different structural domains [22]. Different subtypes of MMPs regulate ECM degradation in unique ways. For instance, MMP-2 and MMP-9 are the primary drivers of tumor invasion and metastasis, while MMP-1 and MMP-3 are linked to inflammatory responses and joint diseases. Thus, it is imperative to scrutinize the subtype differences and specific biological effects of MMPs during their functional and applicational research [23]. In the vascular system, MMPs affect the functions of ECs and VSMCs, as well as their migration, proliferation, ${\mathrm{Ca}}^{2+}$ signaling and contraction. It has been reported that during arterial and small artery remodeling under pressure, from the earliest changes in wall thickness to the formation of aneurysms, MMPs are required [24], and MMPs are also associated with the formation of varicose veins [25]. Research has shown that MMPs play a crucial role in various physiological conditions such as connective tissue remodeling, cell proliferation and differentiation, embryonic development, angiogenesis, and apoptosis [26]. However, activated MMPs can lead to various complications including VSMC proliferation, changes in cell adhesion, vascular permeability, and disarrangement of elastin and collagen proteins. Moreover, the degradation of ECM components after MMPs are activated can promote hypertension [27]. The literature suggests that MMPs are a significant risk factor for complications of hypertension like stroke, atherosclerosis, kidney disease, heart disease, and aneurysms, and contribute to cardiac hypertrophy in the hypertensive state and the transition to heart failure [28]. Kalani and his colleagues [29] discovered that administering an MMP inhibitor to salt-sensitive rats intraperitoneally for a duration of four weeks can result in lowered blood pressure. Their study found that MMP inhibition improved the oxidative/nitrosative stress and tight junction proteins in the brain vasculature of these rats. This suggests that inhibiting MMP-9 may have therapeutic benefits in alleviating hypertension and hypertension-related cerebrovascular disease in salt-sensitive patients [29]. In another study, Seim *et al*. [30] conducted research on plasma extracellular matrix (ECM) remodeling markers in patients with heritable thoracic aortic disease (HTAD) by employing an enzyme-linked immunosorbent assay. They found that all subgroups of HTAD patients have elevated levels of MMP-9, indicating its role as a mediator of inflammation and ECM remodeling. Moreover, other studies highlight the significance of MMP-2 in decreasing the translation of calponin-1. This may lead to VSMC phenotypic switching and migration, resulting in inadequate arterial remodeling adaptation in early hypertensive patients [31]. Recent summaries of hypertension-related vascular remodeling mechanisms indicate that increased oxidative stress impairs nitric oxide bioavailability and increases vascular MMP activity [18]. Oxidative stress is a result of an excessive production or inhibition of deactivation of ROS. The increased formation of ROS triggers a cascade of protein oxidation and cellular signaling events. This process ultimately leads to endothelial dysfunction and MMP activation. Another mechanism associated with increased oxidative stress is the reduced bioavailability of endothelium-derived vasodilators. In a study conducted by Rodrigues [32], aortic remodeling, MMP activity, and ROS levels were assessed in *apolipoprotein E* (*${\mathrm{ApoE}}^{-⁄-}$*) and ovariectomized (OVX) mice treated with doxycycline. The findings indicated that the administration of doxycycline resulted in reduced levels of ROS, MMP-2 expression, and activity in *${\mathrm{ApoE}}^{-⁄-}$*/OVX mice, ultimately leading to a decrease in atherosclerotic lesions. This finding suggests that drug therapy may help prevent vascular remodeling associated with MMP activity and expression, while reducing ROS. These new insights may provide opportunities for the treatment of hypertension.

#### 2.1.2 Cathepsin 

Cathepsin serves as a crucial enzyme in reshaping the ECM. Its primary function is to facilitate the breakdown and regeneration of the matrix, thereby preserving the structure and functionality of tissues. As such, cathepsin has a significant impact on physiological and pathological processes, including cell migration, tissue repair, and cell activity. In the vascular system, Cathepsins belongs to the protease family and regulates the ECM under physiological and pathological conditions [33].

The MEROPS database lists over 700 human proteases, with 11 of them being cysteine cathepsins, including cathepsin B, C, F, H, K, L, O, S, V, W, and X. Cysteine cathepsins primarily contribute to the development of cardiovascular diseases, given their ability to degrade components of the ECM, particularly elastin. Cathepsin K, S, and V are capable of degrading elastin, which plays a crucial role in maintaining vascular integrity. Excessive elastin degradation, often associated with atherosclerosis, aneurysm, and chronic kidney disease [34, 35], can lead to a weakened vascular structure and rupture. It has been confirmed that overexpression of cathepsin S induces pulmonary artery remodeling [34]. Studies in the cardiovascular field have highlighted that vascular cells associated with atherosclerotic lesions overexpress cathepsin S without corresponding changes in cystatin C expression. This suggests that the balance between cathepsin and its inhibitors has shifted, facilitating cardiovascular wall remodeling. Further research indicates that the upregulation of cathepsin S in vascular disease can impede the integrity of the elastic layer and the basal layer of the intima. Microvessel growth is negatively impacted by cathepsin S inactivation, but does not impact vascular endothelial growth factor (VEGF) and basic fibroblast growth factor expressions. Overall, cathepsin S promotes ECM degradation. In addition to cathepsin S, cathepsin K is also considered an essential marker of vascular remodeling. It inhibits vascular smooth muscle cell proliferation and thus the vascular remodeling process [36]. It has been demonstrated that patients with endothelial dysfunction in chronic kidney disease have shown significant increase in the expression level of cathepsin D [37].

The Cathepsin L genes encode two different cathepsin proteins, namely cathepsin L and Cathepsin V. In a study by Lu *et al*. [38], it was found that vascular remodeling induced by Ang II as well as hypertension were mediated by mechanisms relating to cathepsin L/V-MEK/ERK. This confirmed that cathepsin proteins and related factors such as cystatin C and mitogen-activated protein kinase phosphorylation were up-regulated in mesenteric arteries and serum in hypertensive patients. Furthermore, cathepsin proteins are known to play a key role in the degradation and transfer of ECM. In an experiment involving the knockout of cathepsin proteins, Pan *et al*. [39] found a significant increase in collagen deposition in the medial aorta layer of cathepsin proteins knockout mice, while telomerase activity analysis suggested changes in vascular aging in cathepsin proteins knockout mice. In addition, a decrease in cathepsin proteins was observed in aging ECs [40]. Enhanced expression of A2, a member of the aldehyde dehydrogenase 1 family, was also reported. Activation of the AKT/ERK1/2-P21 pathway was found to promote cellular senescence, which may play a critical role in vascular senescence. These findings indicate that cathepsin proteins hold great potential as a therapeutic target for treating EC senescence. Yu *et al*. [41] revealed that elevated levels of cathepsin proteins in plasma were closely linked to coronary artery disease, providing evidence that circulating cathepsin proteins could be a promising biomarker for this condition. Furthermore, research on human atherosclerotic lesions and narrowed aortic valves has shown that, apart from cathepsin K and S, there is a notable increase in both mRNA and protein expression levels of cathepsin V [42]. Additionally, previous research has highlighted that the degradation of elastic fibers by cathepsin V, K, and S aids in the development of plaque-associated vascular calcification in VSMC [43]. As a result, cathepsin plays a crucial role in the process of vascular remodeling.

### 2.2 The Impact of Angiotensin on Vascular Remodeling

The RAS system, vascular remodeling, and hypertension are all interconnected through a complex network, with Ang II playing a significant regulatory role. Ang II is a multifunctional hormone that is produced by the RAS system. In the kidney, renin is released by the ECs of the glomerulus, which break down plasma angiotensinogen to generate a peptide precursor called angiotensin I. This is then transformed into Ang II by angiotensin-converting enzyme (ACE). Ang II plays a pivotal role in regulating hormonal responses in the body, such as the release of adrenaline and noradrenaline from the hypothalamus and adrenal medulla, which regulate water-sodium balance and the activity of the sympathetic nervous system. When Ang II binds to the Ang II type1/AT1 receptor (AT1R), it triggers an interaction between AT1R and heterotrimeric G proteins. This then leads to a cascade of second messenger signaling, involving key molecules such as inositol trisphosphate, diacylglycerol, arachidonic acid, and ROS. As a result, downstream effectors, including phospholipases C, A, and D, are activated. Furthermore, Ang II can stimulate inflammatory reactions, cell proliferation and differentiation, and contribute to various physiological and pathological processes such as cardiac hypertrophy and hypertension. Therefore, abnormal regulation of Ang II can affect the progression and treatment of hypertension, and other cardiovascular diseases.

One of the primary ways that Ang II contributes to vascular remodeling is by increasing oxidative stress [19]. The resulting oxidative stress may trigger the activation of MMPs, which can cause the deterioration of ECM and subsequently lead to vascular remodeling. Ang II increases oxidative stress by activating Nox to produce ROS [44]. Ang II is thought to enhance the generation of ROS by upregulating the expression and catalytic activity of the Nox family proteins [45]. The abundance of proteins of Nox family isoforms in VSMC will determine the physiological function of Ang [46]. Nox2 and Nox4 are primarily found in endothelial cells and cardiomyocytes, indicating their close association with cardiovascular diseases [47]. This aligns with the notion that the expression of Nox subunits in the vascular wall plays a crucial role in the microvascular remodeling observed in individuals with arterial hypertension. For example, Sortilin is a member of the vacuolar protein sorting 10 protein (VPS10P) receptor family. There is evidence suggesting that sortilin plays a key role in the dysregulation of sphingolipid metabolism and oxidative stress associated with human hypertension. Studies have found increased plasma acid sphingomyelinase (ASMase) activity, as well as elevated levels of plasma sortilin, sphingosine-1-phosphate receptor (S1P), and soluble Nox2-derived peptide (sNox2-dp) in hypertensive patients, with a more pronounced increase in those with uncontrolled blood pressure. Sortilin induces dysfunction of the mesenteric arterial endothelium through the activation of the Nox2 isoform, and this dysfunction can be prevented by lowering ASMase or sphingosine kinase 1 [48] Nox2 induces vascular oxidative stress by directly generating superoxide, whereas Nox4 primarily relies on the swift conversion of superoxide into hydrogen peroxide (H2O2) through dismutation [49].

Another main way through which Ang II contributes to vascular remodeling is by promoting inflammation in the vascular wall [19]. This is achieved by activating various pro-inflammatory cytokines and chemokines, leading to the recruitment of leukocytes and activation of resident immune cells. This, in turn, further drives vascular remodeling, as discussed in Section 3.2. Ang appears to be central and is able to activate and/or induce multiple factors involved in vascular fibrosis, to stimulate collagen fiber hyperplasia and ECM deposition by inducing the expression of connective tissue, growth factors, inflammatory factors, aldosterone and ET-1 [50, 51]. Consequently, this leads to the accelerated growth and development of VSMCs, ultimately promoting vascular remodeling. Additionally, Ang II triggers integrins, adhesion molecules, cytokines, and fibrosis, ultimately promoting inflammation and remodeling in the vascular system. Endothelial remodeling due to Ang II can induce both growth and apoptosis in cells, leading to a reduction in the outer diameter of blood vessels. However, it is important to note that the apoptosis is limited only to the periphery [20, 52]. A recent study has demonstrated the crucial role of AT1R expression in VSMCs in vascular remodeling within a mouse model of hypertension induced by Ang II infusion [53]. The underlying mechanism by which Ang II promotes vascular fibrosis and arterial stiffness should be highlighted. This process involves the interaction between adventitial fibroblasts, VSMC, immune cells, and ECM with inflammatory mediators and associated signal transduction pathways [20].

### 2.3 Stem Cells and Vascular Remolding

Stem cells are a type of highly potential cell that have unlimited self-renewal ability and multi-directional differentiation potential. Recent studies have demonstrated that vascular resident stem cells, which are present in the tunica intima, tunica media and tunica externa, play an essential role in cellular regeneration. The composition and distribution of vascular resident stem cells are key factors in the complex structure of arteries. The innermost layer, also referred to as the endothelial cell layer, contains a small population of endothelial progenitor cells (EPC) [54, 55], as well as notable stem cells such as Sca-1+ and CD34+. Meanwhile, the middle layer serves as a thick layer composed of VSMCs with a small population of stem cells such as Sca-1+ stem cells. Finally, the outer layer comprises a layer of connective tissue, and is home to a diverse group of cells, including fibroblasts, resident inflammatory cells, peri-vascular endothelial cells, adrenergic nerves, as well as stem cells (like pluripotent stem cells or bone marrow mesenchymal stem cells), and progenitor cells (including cells with differentiation potential for macrophages, endothelial cells, smooth muscle, and hematopoietic cells like Sca-1+, CD34+, c-Kit, and Flk-1, among others). These blood vessel stem/progenitor cells are spread throughout the entire structure of the vessel wall and are vital in the development of vascular diseases, as well as considered as the cell source for blood vessel remodeling.

A recent genetic cell lineage tracing study [56] showed that c-kit+ cells can be transplanted into blood vessels in animals and differentiate into VSMCs. Experiments on cell culture *in vitro* also support that these cells have stem/progenitor properties. Meanwhile, other studies [3] have confirmed that c-kit+ stem cells differentiate into vascular cells in small blood vessels. Another study using genetic lineage tracing technology in lung maintenance and repair processes has confirmed that c-kit+ cells was involved in the formation of pulmonary vascular endothelium [57]. Related studies have shown that CD146+ cells constitute the majority of embryonic aortic VSMCs, and rapid regeneration of the smooth muscle layer is crucial for successful repair of vascular injury. Jiang *et al*. [58] conducted single-cell RNA sequencing (sRNA-seq) on mouse femurs and found that the percentage of CD34+ expression showed a notable increase in the femoral arteries where lesions were detected. A series of data showed that most lumen and microvascular CD31+ endothelial cells were derived from non-bone marrow CD34+ cells to respond to vascular injury, while bone marrow CD34+ cells mainly caused an increase in CD45+ white blood cells, which may also be an important factor in neo-intima formation. Structural atherosclerosis, a well-established cardiovascular risk factor, is dependent on hematopoietic stem cells, specifically CD34+ cells. However, it is noteworthy that there is a negative association between the number of circulating CD34+ cells and cardiovascular disease. In a recent epidemiological study comprising Japanese men aged 60 to 69 years attending annual health examinations, Shimizu identified [59] that functional atherosclerosis differs significantly from structural atherosclerosis in terms of endothelial repair activity. Aggressive endothelial repair may result in an increase in both functional and structural atherosclerosis. The depletion of CD34+ cells leads to endothelial repair defects, which further exacerbates functional atherosclerosis instead of structural atherosclerosis. Therefore, the absence of structural atherosclerosis does not always indicate a favorable condition for the endothelium. Tang *et al*.’s [60] genetic lineage tracing and single-cell RNA sequencing found that after severe endothelial damage, Sca-1+ VSMCs migrate to the middle layer and generate new VSMCs, which have a greater tendency to expand compared to existing VSMCs, and are more effective in subsequent expansion than prior existing smooth muscle. Their study determined that Sca-1+ PDGFRa+ cells are a source of new smooth muscle healing after severe cross-cut artery injury. Research has shown that upon acute vascular injury, Sca1+ cells differentiate into myofibroblasts and are embedded in perivascular collagen and ECM. They found that the chromatin remodeler, Smarca4/Brg1, facilitates AdvSca1-SM myofibroblast differentiation [61].

Ion channels play a key role in various aspects of cell function, including pulse excitation and propagation, proliferation, migration, and apoptosis. The role of ion channels in vascular remodeling will be discussed in detail in the following Section 3.3. For stem cells, the importance of ion channels in cell proliferation, migration, and differentiation is increasingly recognized [62]. Research has shown that various membrane ion channels and pump proteins can effectively transport ions across lipid bilayers, establish membrane potential (Vm), and generate sustained signals that are more persistent than excitatory cell action potentials. This self-regulating bioelectric signal not only reflects the state of the cell, but also controls various characteristics of progenitor cells [63, 64]. Previous studies have suggested that the regulation of Vm can serve as an upstream factor influencing stem cell differentiation and can impact downstream processes, ultimately influencing the expression of genes related to progenitor cell maturation [65]. However, research on the relationship between stem cells and vascular remodeling is still limited. Further research will help elucidate the functional role of ion channels in stem cells and their role in vascular remodeling.

## 3. Vascular Remodeling and Hypertension

### 3.1 The Connection between High Blood Pressure and Vascular Remodeling 

Hypertension is a chronic disease that is widely prevalent and characterized by elevated arterial blood pressure. It is defined by systolic and/or diastolic pressure readings ≥140 mmHg and ≥90 mmHg, respectively, and often leads to functional and organic damage in vital organs such as the heart, brain, and kidneys. Research suggests that hypertension is primarily a result of increased peripheral blood pressure caused by the rise in smooth muscle tone of arteriolar walls and heightened reactivity to vasoactive substances like catecholamines, 5-hydroxytryptamine, and Ang Ⅱ, along with structure changes in vascular constriction. It is important to emphasize that an increase in sympathetic activity is widely acknowledged as a crucial factor in the development and maintenance of high blood pressure, as well as a significant contributor to vascular remodeling in individuals with hypertension [9]. Numerous studies have demonstrated the link between sympathetic hyperactivity and the occurrence of hypertension and metabolic syndrome. For instance, sympathetic hypertension has been associated with elevated hematocrit levels and excessive platelet aggregation, which can promote the development of coronary heart disease [66]. Furthermore, heightened sympathetic activity leads to an increased rate of left ventricular systolic contraction, heightened arterial tone, reduced aortic compliance, and enhanced constriction of arterioles, all of which have a direct impact on systolic blood pressure [67]. Essential hypertension is characterized by heightened sympathetic activation in skeletal muscle vessels, heart, and kidneys, particularly among young individuals. Several studies have provided compelling evidence of widespread autonomic abnormalities in the initial stages of hypertension, with excessive sympathetic activity observed in these patients since childhood [68]. In summary, numerous reports [10, 66, 69, 70, 71, 72] have focused on the correlation between hypertension and heightened sympathetic nerve activity. However, due to space limitations, this article will not delve into this aspect.

Prolonged high blood pressure can lead to vascular remodeling, including changes in the structure of blood vessels such as thickening of vessel walls and reduced distensibility. It also triggers abnormal growth, differentiation, migration, synthesis, and secretion of vascular tissue cells. Furthermore, disruptions in ion channel function may interfere with the normal regulation of blood vessel dilation and constriction. After suffering vascular injury, the vessel wall structure may undergo significant modifications, with intimal regeneration being a critical component in the healing process. Hypertension patients’ increased systemic vascular resistance is likely due to vascular remodeling being the leading cause. Changes to the blood vessels due to hypertension are complex and closely related to the fluctuations in hormone and vasoactive substance levels. Humphrey *et al*. [73] emphasize the importance of exploring the mechanism of hypertension-related vascular remodeling from a mechanical homeostatic perspective. Elevated blood pressure is the root cause of a series of arterial responses triggered by phenotypic changes in primary vascular wall cells and differential gene expression, leading to vascular remodeling. Aortic maladjustment is a condition characterized by fibrosis of the outer membrane, which greatly reduces its biomechanical function, resulting in impaired end-organ perfusion. This leads to an increase in the incidence and mortality of related diseases. The predominant components found in the walls of blood vessels are collagen and elastin, and the stiffness of arteries is determined by their presence [74]. Excessive deposition of collagen can damage the walls of blood vessels, leading to increased stiffness. The hypertrophy, sclerosis, and apoptosis of VSMCs contribute to the thickening of the inner layer of blood vessels and the stiffness of arteries. Studies have demonstrated that the increased stiffness of vascular ECM during hypertension activates stroma-binding receptor integrins and associated intracellular signaling pathways, including phosphatidylinositol 3-kinase/protein kinase B, beta-catenin, and RhoA/Rho-associated protein kinases. As a result, VSMCs undergo contraction, proliferation, and migration [75]. Elastin, a component of the ECM, also plays a crucial role in regulating VSMC behavior and the transition of their phenotype to a contractile state [76].

### 3.2 Inflammatory Factors and Their Role in Hypertension-Induced Vascular Remodeling

Recent research has highlighted the crucial role of an activated inflammatory response and immune system in the development and progression of hypertension [17] as well as its association with various complications. In individuals diagnosed with high blood pressure, researchers have observed increased levels of inflammatory biomarkers, suggesting the potential involvement of the immune system in the development of hypertension. Furthermore, inappropriate immune activation may contribute to high blood pressure by impacting different organs like the microvasculature, kidney, and nervous system. It is widely known that an imbalanced and overactive immune system can cause inflammation, which is characterized by a surge of proinflammatory cytokines. High blood pressure triggers the body’s natural inflammatory response, which leads to the accumulation of white blood cells and the release of inflammatory factors. These factors can build up under the lining of blood vessels, resulting in further thickening of the walls and the formation of plaques [17]. Although inflammation serves as a critical response of the body to foreign agents and promotes healing, an excessive presence of inflammation can be detrimental. Inflammation and endothelial dysfunction are intimately connected and contribute to every stage of injury and thrombotic complications. According to a study by Adachi *et al. * [77], following vascular guidewire injury, the expression of inflammatory cytokines like TNF-α, Serpine1 (PAI-1), IL1α, and IL1β in the surrounding adipose tissue significantly increased. Another study demonstrated that flow cytometry with 15 parameters was utilized to evaluate the expression of leukocytes and 13 subtypes of leukocytes at four different time points subsequent to femoral artery wire injury. The findings indicated the significance of the CD64(+)Tim4(+) (Leukocyte differentiation antigen positive and T cell immunoglobulin and mucin domain 4 positive) macrophage phenotype expression during the initial seven days post-injury. Most components of the vascular system are delivered through blood vessels and play an essential role in the body’s inflammatory response. According to a study by Zanoli *et al*. [78], inflammation can have harmful effects on arterial physiology and lead to severe vascular complications. This inflammatory process can also trigger endothelial dysfunction, which can have detrimental consequences for the cardiovascular system. A defining characteristic of essential hypertension is the notable upsurge in cytokine and chemokine-induced oxidative stress. Intriguingly, this inflammatory process is intricately linked to the generation of ROS. Endothelial cells, monocytes, and macrophages generate abundant quantities of superoxide anions (•O2) and H2O2, contributing to a state of mild inflammation that fosters oxidative stress and subsequently impairs vascular function. One particular study investigated the effect of interleukin-17A (IL-17A) on Ang II-induced endothelial injury in the context of hypertension. The study found that neutralizing IL-17A or specific inhibition of the IL-17A receptor prevented Ang II-induced neurovascular coupling damage and brain •O2 production. Their study reported that the long-term use of IL-17A can harm neurovascular endothelial cells and increase •O2 production [79].

Traditionally, it was thought that essential hypertension was caused by changes in hemodynamics. However, numerous studies have unequivocally confirmed that inflammatory cytokines also hold a crucial role in promoting the progression of hypertension through their impact on blood vessels and kidney function. One such cytokine is IL-1β, which belongs to the IL-1 family of interleukins that are known to be crucial in inflammation and disease. When IL-1β combines with the IL-1R1 receptor and its associate protein IL-1RAcP, it promotes the up-regulation of multiple genes, including IL-6, IL-17, IFN-g, and IFN-γ. These genes trigger downstream processes of pro-inflammatory cell events linked to disease progression and tissue damage. The activity of IL-1β can be inhibited by competitive binding of IL-1Rα to IL-1R1 [80, 81]. Studies have demonstrated that IL-1β is directly involved in triggering the inflammatory response and influencing the phenotype and function of VSMCs through inflammation-dependent or independent mechanisms, ultimately leading to vascular remodeling. For instance, a research study assessing the impact of the IL-1R1 receptor inhibitor Anakinra on reducing blood pressure in obese patients revealed that Anakinra significantly decreased systolic blood pressure and peripheral vascular resistance [82]. This finding supports previous evidence suggesting the potential involvement of IL-1β in the development of hypertension. In another study focused on salt-sensitive hypertension, it was discovered that IL-1β released from renal tubular epithelial cells in diabetic mice can promote hypertension by polarizing macrophages into the M1 subtype, resulting in the excessive release of IL-6. However, this effect can be attenuated by inhibiting the synthesis of IL-1β in immune cells or knocking out the IL-1 R1 receptor [83]. Therefore, targeting IL-1β may offer a promising treatment strategy for hypertension. Furthermore, there is compelling evidence linking IL-17 to elevated blood pressure. In an animal model of Ang II-induced hypertension, plasma IL-17 levels were found to be significantly elevated. Conversely, inhibition of IL-17 was associated with reduced blood pressure and decreased collagen deposition in the heart and kidneys. *In vitro* experiments have shown that IL-17 promotes the expression of cytokines and chemokines in human VSMCs, recruits more inflammatory cells, and decreases the production of NO by phosphorylating threonine 495 of eNOS in aortic endothelial cells [84]. A growing body of evidence strongly supports a positive correlation between IL-6 levels and blood pressure. Animal studies, for instance, have demonstrated that *in vivo* injection of Ang II can increase plasma IL-6 concentration and blood pressure levels. Conversely, in IL-6 knockout mice, the pressor effect of Ang II is significantly diminished [85].

TNF-α is an established inflammatory cytokine renowned for its involvement in the acute phase response. Numerous studies conducted on both humans and rodents have shown an elevation in TNF-α expression in cases of hypertension. The activation of TNF-α receptors has been linked to various effects such as apoptosis, Nox activation, and the activation of nuclear factor κB (NFκB). Research suggests that NFκB and Nox activation promote hypertension by increasing the expression of chemokines and adhesion molecules in blood vessels, ultimately leading to microvascular remodeling. It is believed that a subset of gammadelta T cells, which produce IL-17A, may contribute to the progression of hypertension. The development of these cells is dependent on IL-23 receptor (IL-23R) stimulation. One study has confirmed that the elevation of blood pressure and vascular injury induced by Ang II can be reduced in IL-23R knock-in mice that lack functional IL-23R. Moreover, the results indicate that the deficiency of functional IL-23R is associated with an increase in IFN-γ-producing T cells and an exaggerated development of Ang II-induced hypertension. This effect is partly mediated by IFN-γ [86]. IFN-γ, as a critical member within the type II interferon family, serves a significant function in adaptive immune responses. Studies have consistently demonstrated that IFN-γ plays a significant role in promoting inflammation and oxidative stress, ultimately giving rise to hypertension. Furthermore, the production of ROS induced by IFN-γ poses a detrimental impact on blood vessels, exacerbating the development of hypertension. Another noteworthy mechanism through which IFN-γ may contribute to hypertension involves its influence on RAS. In fact, research has illustrated that IFN-γ triggers hypertension by heightening angiotensinogen expression in rat proximal renal tubule cells in a JAK2/STAT3-dependent manner [87], thereby leading to an augmented blood volume. However, according to Ishimitsu *et al*. [88], IFN-γ ameliorated the development of hypertension and vascular and renal injuries in Dahl salt-sensitive rats. The resolution of vascular and renal injuries contributes, in part, to the antihypertensive action of IFN-γ.

In addition to cytokines, integrins also play a significant role in inflammation. Budatha *et al*. [89] conducted a study on integrin α5/2 mice and discovered that they exhibited reduced fibronectin deposition and decreased inflammatory activation of endothelial cells in acute hypertension. Using the transverse aorta constriction (TAC) technique to induce high blood pressure, the results showed that the mutation of integrin α5/2 in mice can effectively counteract the inflammatory effect of fibronectin, leading to decreased arterial wall thickening and significantly affecting vascular remodeling. Recently, Lin *et al*. [90] have provided further evidence confirming the role of integrin CD11b in hypertension and vascular dysfunction. Their study highlights the crucial involvement of CD11b in mediating macrophage adhesion and migration, thereby contributing to the development of hypertension and vascular dysfunction. This study emphasizes the significance of CD11b+ myeloid cells in promoting these conditions. The inflammasomes, which are composed of NOD-like receptor (NLR)-family pyrin domain-containing proteins like NLRP1 and NLRP3, are cytosolic protein complexes expressed primarily in innate immune and endothelial cells. NLRP1 and its downstream molecules ASC (adaptive signal transduction molecules) can activate pro-inflammatory factors IL-1β and IL-18 through a caspase-1-dependent pathway, thereby aggravating vascular inflammation and leading to the occurrence of hypertension. Recent studies have uncovered a connection between the activation of the NLRP3 inflammasome and endothelial dysfunction [91]. Notably, it has been suggested that deficiencies in NLRP3 can provide significant protection by preventing the breakdown of tight junctions and reducing endothelial permeability. Moreover, pharmacological inhibition of the NLRP3 inflammasome has displayed promising therapeutic effects. Recent research has shed light on the potential advantages of NLRP3 knockdown, including the reduction of blood pressure, improvement of vascular remodeling, alleviation of insulin resistance, and the postponement or improvement of atherosclerosis through metabolic regulation, relief of oxidative stress, and reduction of cytokine-induced inflammation [92, 93].

### 3.3 Hypertensive Vascular Remodeling and Ion Channels in Vascular Cells

Ion channels are a crucial component of the cell membrane, comprising specialized proteins that are expressed on the surface. These channels, including ${\mathrm{Ca}}^{2+}$, $K^{+}$, ${\mathrm{Na}}^{+}$, ${\mathrm{Cl}}^{-}$, and so on, are especially important in vascular cells, playing a significant role in regulating cell membrane potential, cell signaling, vascular contraction function, and maintaining vascular homeostasis. Interestingly, research has linked diseases such as hypertension, coronary heart disease, and stroke to increased peripheral vascular resistance or spasms, resulting from abnormal contraction of resistance arteries and small arteries caused by the abnormal function and expression of channels in VSMCs. In addition, studies have shown that “ion channel remodeling” linked to vascular spasm in coronary heart disease may be linked to vascular dilation and contraction dysfunction. Not only do ion channels impact smooth muscle function, but they also play a crucial role in sustaining the function of other vascular cells. Recently, stem cell biology has also highlighted the critical role of membrane ion channels. Ion channels regulate membrane potential and excitability in excitable cells, muscle contraction, and exocytosis by controlling ion flux. In non-excitable cells, they control cell volume, polarity, acidity, and various other cellular processes.

Vascular tone is regulated by a variety of factors, including neural and humoral stimuli. VSMCs integrate these signals and adjust the contractile state of VSMCs by modulating intracellular calcium levels ([${\mathrm{Ca}}^{2+}$]_i_) through ${\mathrm{Ca}}^{2+}$ influx through membrane channels and ${\mathrm{Ca}}^{2+}$ release from intracellular stores. This process effectively regulates vascular diameter and resistance in order to meet the specific needs of tissue activity.The primary function of smooth muscle contraction is the entry of ${\mathrm{Ca}}^{2+}$ through membrane channels, while the role of $K^{+}$ channels is to act as negative regulators for relaxation. Upon opening, $K^{+}$ channels increase $K^{+}$ efflux, membrane potential hyperpolarizes, and voltage-gated ${\mathrm{Ca}}^{2+}$ channels close, resulting in vasodilation. Furthermore, $K^{+}$ channels play a role in determining vascular tone and diameter by regulating smooth muscle cell membrane potential. Research shows that voltage- and ${\mathrm{Ca}}^{2+}$-sensitive large conductance $K^{+}$ channels (i.e., ${\mathrm{BK}}_{\text{Ca}}$ channels) are the principal membrane proteins maintaining vascular tone in smooth muscle. Changes in ${\mathrm{BK}}_{\text{Ca}}$ may be caused by genetic mutations or compensatory mechanisms due to the disease-induced gene expression changes. Studies on mesenteric arteries in Chinese Han hypertensive patients have found that reduced ${\mathrm{BK}}_{\text{Ca}}$ channel activity in VSMCs during hypertension results from the downregulation of the channel’s β1 subunit gene and protein expression [94, 95, 96].

In addition to VSMCs, ion channels present in other cells of blood vessels also play crucial roles in maintaining proper vascular function. For instance, it was found that hypertension-induced vascular damage is caused by an increased production of ROS and altered ${\mathrm{Ca}}^{2+}$ handling. Transient receptor potential melastatin 2 (TRPM2) is a regulator of ROS sensing and ${\mathrm{Ca}}^{2+}$ and ${\mathrm{Ca}}^{+}$ transport, and serves as a cross-talk point between redox and ${\mathrm{Ca}}^{2+}$ signaling in VSMCs. Researchers have conducted studies in vitro, using ECs from arteries of normal and hypertension-induced individuals, as well as wild-type and hypertension mice (LinA3). Results have proven that TRPM2 activation, regulated by ROS-dependent poly [ADP-ribose] polymerase 1 (PARP1), promotes ${\mathrm{Ca}}^{2+}$ and ${\mathrm{Na}}^{+}$ influx in blood vessels through the ${\mathrm{Na}}^{+}$/${\mathrm{Ca}}^{2+}$ exchanger (NCX). This suggests that oxidative stress-induced upregulation of this pathway may be a newly discovered participant in hypertension-related vascular dysfunction. The epithelial sodium channel (ENaC) plays a critical role in regulating extracellular fluid and blood pressure [97]. Recent research indicates that aside from its responsibility for ${\mathrm{Na}}^{+}$ handling in the kidney, ENaC expressed in other cells, including immune cells, can influence blood pressure. Dendritic cells (DCs) play a crucial role in salt-sensitive hypertension and are activated in an ENaC-dependent manner. A study has highlighted the importance of extra-renal ENaC regulation in salt-sensitive hypertension as a potential novel therapeutic target [98]. Moreover, studies have shown that overexpression of ENaC in epithelial cells correlates with high arterial pressure and can serve as a biomarker for detecting this disease [99]. Diabetic nephropathy is associated with hypertension, proteinuria, and urinary fibrinolysis protein excretion that is activated by ENaC *in vitro*. Some researchers studied the protective effects of plasminogen deficiency and amiloride treatment on hypertension in diabetic mice. They suggested that plasminogen may promote hypertension in diabetes with proteinuria through ENaC [100].

ECs release various vasodilating factors, including nitric oxide and prostacyclin, in response to stimulatory and shear stress. Additionally, vascular ECs are known to regulate VSMC contractility through the production of endothelium-dependent hyperpolarization (EDH). The opening of small and intermediate conductance calcium-activated potassium channels (${\mathrm{SK}}_{\text{Ca}}$ and ${\mathrm{IK}}_{\text{Ca}}$ channels) is thought to be the initial mechanism in EDH generation. In hypertension, EDH and EDH-mediated relaxation are impaired in animal models and humans. However, anti-hypertensive therapy has been shown to restore normal EDH function. Recently, scholars have proved that endothelial ${\mathrm{IK}}_{\text{Ca}}$ and ${\mathrm{SK}}_{\text{Ca}}$ channel activation decreases pulmonary arterial pressure and vascular remodeling in pulmonary hypertension [101].

Piezo1 and Piezo2, two recently discovered mechanosensitive channels, combine fine force sensing with regulated ${\mathrm{Ca}}^{2+}$ influx by forming a transmembrane triangle. There is increasing evidence that the transmembrane triangle plays an important role in endothelial shear stress sensing and secretion, NO generation, vascular tone, angiogenesis, atherosclerosis, vascular permeability and remodeling, blood pressure regulation, and pressure receptor reflex [102]. The voltage-dependent L-type ${\mathrm{Ca}}^{2+}$ ion channels are well established in ${\mathrm{Ca}}^{2+}$ influx and their role in the development of cardiovascular diseases. However, over the past 20 years, another type of ${\mathrm{Ca}}^{2+}$ channel, voltage-independent store-operated ${\mathrm{Ca}}^{2+}$ channels (SOCE), has been gradually recognized for their involvement in ${\mathrm{Ca}}^{2+}$ entry regulation and fine-tuning in cardiac and VSMCs. These channels are controlled by STIMs and Orai channels, whose protein alterations are believed to facilitate ${\mathrm{Ca}}^{2+}$ entry, and thus promote the development of cardiovascular dysfunction. The development of selective ${\mathrm{Ca}}^{2+}$ channel inhibitors presents challenges in improving hypertension treatment [103]. Wang *et al*. [104] reported that during atherosclerosis, down-regulation of SOCE components and impaired eNOS activity can reduce SOCE-induced endothelial progenitor cell dysfunction.

**Fig. 2.**
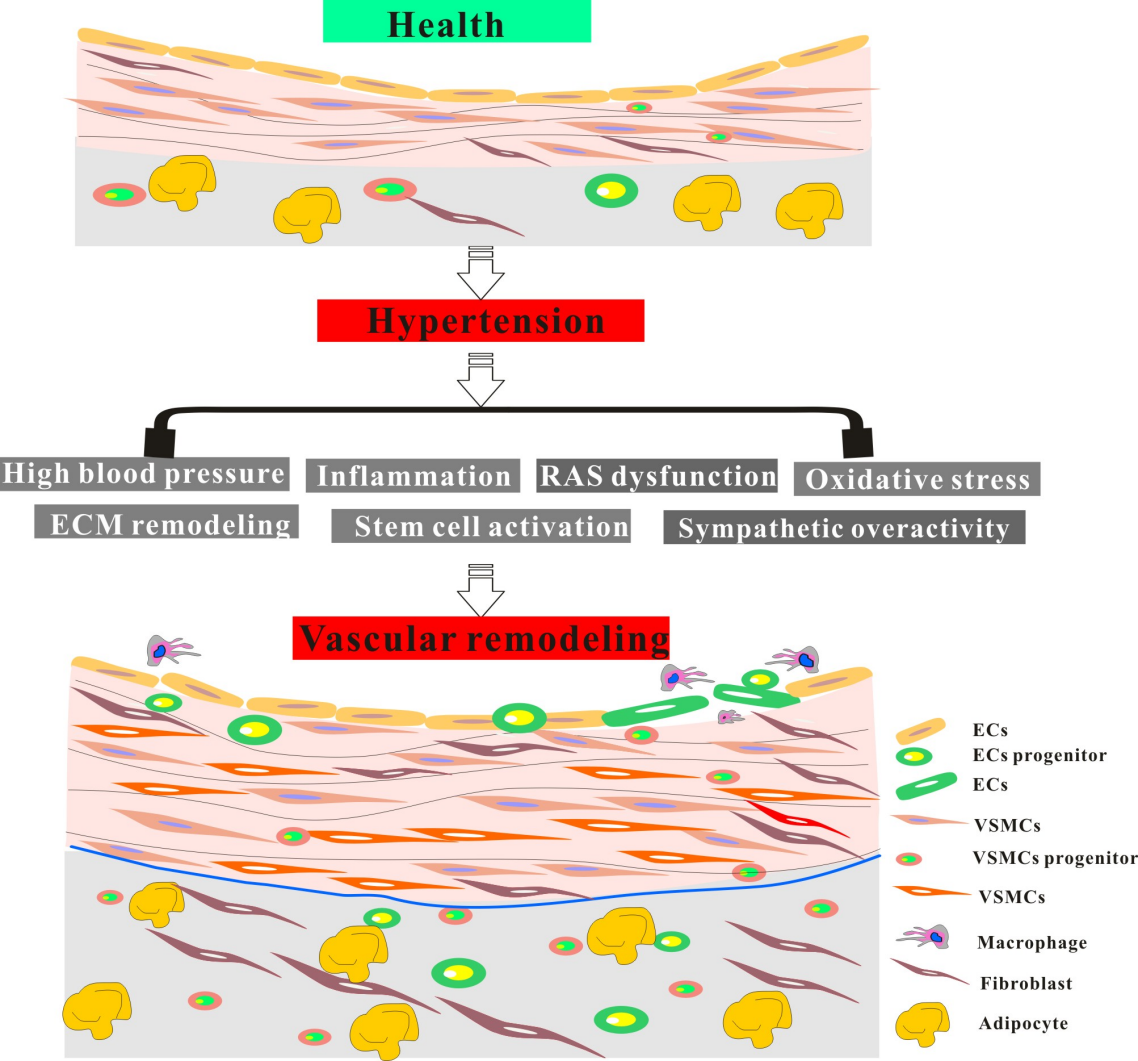
**The key factors and crucial mechanisms of vascular remodeling under hypertension**. The abnormal activity of major factors in hypertension leads to severe vascular remodeling, further exacerbating vascular injury and hypertension. RAS, renin-angiotensin system; ECM, extracellular matrix; ECs, endothelial cells; VSMCs, vascular smooth muscle cells.

Fig. 2 illustrates the critical factors and innovative mechanisms involved in vascular remodeling in hypertension.

## 4. Conclusions

Hypertension is a widespread cardiovascular disease that has a considerable impact on human health. While the pathogenesis of this disease is intricate, it is clear that it is intimately linked to vascular remodeling. Researchers are currently exploring different mechanisms that contribute to vascular remodeling in hypertension, such as changes in the ECM, inflammatory mechanisms, stem cell involvement, and ion channel involvement. However, the specific interactions between vascular structure and the resulting functional changes that contribute to the pathogenic effects of hypertension need further investigation. It is widely acknowledged that vascular structure and function modifications can exacerbate the effects of hypertension, and the underlying mechanisms are gradually being uncovered. These research findings offer valuable insights into the identification of new biomarkers, innovative therapies, and targets for treatment of treatment of hypertension and other cardiovascular diseases. In this review, we summarize the latest research progress of RAS, inflammation, ECM, and stem cells in vascular remodeling. We also discuss the latest research on ion channels found in vascular cells and stem cells. A comprehensive understanding of hypertension and vascular remodeling will undoubtedly fuel more research, leading to the discovery of more effective treatment methods and improved measures for disease prevention and control. Therefore, we hope that this summary of current knowledge will serve as a significant stimulus for future research in the field.
